# Changes in binge eating symptoms following an online community-based ultra-processed food addiction intervention: Liberate

**DOI:** 10.3389/fpubh.2026.1807450

**Published:** 2026-05-21

**Authors:** Ellen Bennett, Erin L. Bellamy, Deborah Lycett, Jen Unwin, Maxine Whelan, David A. Wiss, Riya Patel

**Affiliations:** 1 Coventry University, Coventry, United Kingdom; 2 Public Health Collaboration, London, United Kingdom; 3 University of East London, London, United Kingdom; 4 Collaborative Health Community, Oxford, United Kingdom; 5 Nutrition in Recovery, Los Angeles, CA, United States; 6 University of Leicester, Leicester, United Kingdom

**Keywords:** abstinence, behavioural change, Binge Eating Disorder, binge eating scale, food addiction, low-carbohydrate diet, relapse prevention, ultra-processed food addiction

## Abstract

**Background:**

Binge Eating Disorder (BED) is the most prevalent eating disorder worldwide and is associated with significant psychological distress, metabolic risk, and high relapse rates. Standard treatments such as Cognitive Behavioural Therapy and pharmacotherapy offer modest long-term efficacy. Increasing evidence links binge eating behaviours with consumption of ultra-processed foods (UPFs), industrial formulations engineered for hyper-palatability that can activate dopaminergic reward pathways in ways similar to addictive substances. The construct of ultra-processed food addiction (UPFA) provides a conceptual framework for understanding this overlap and exploring addiction-informed treatment models.

**Objective:**

To evaluate changes in binge eating symptom severity following participation in Liberate, an online, community-based psychoeducational intervention integrating addiction-informed principles with an abstinent, real-food, low-carbohydrate dietary approach.

**Methods:**

This secondary analysis pooled data from two cohorts of adults (*N* = 117) with self-reported UPFA enrolled in the Liberate feasibility and acceptability study. Binge eating was measured using the Binge Eating Scale (BES) at baseline, post-intervention (8 weeks), and 6-month follow-up. Repeated measures ANOVA assessed changes in continuous BES scores using an intention-to-treat approach with baseline carried forward. Changes in clinical severity categories (none, moderate, severe) were also examined descriptively.

**Results:**

Mean BES scores significantly decreased from 26.5 (95% CI 24.9–28.1) at baseline to 18.0 (95% CI 16.1–20.0; *p* = <0.001) post-intervention, with improvements largely maintained at six-month follow-up (19.2; 95% CI 17.2–21.3; *p* = <0.001 baseline to post; *p* = 0.276 post to follow-up). The proportion of participants with severe binge eating halved post-intervention (48.7 to 24.8%) and remained lower at 6 months (30.8%). While, those with no binge eating increased from 18.8 to 54.7% post-intervention and 48.7% at follow-up.

**Conclusion:**

Participation in an addiction-informed, real food, psychoeducational intervention was associated with significant reductions in binge eating symptoms which were largely maintained over 6 months, with no evidence of worsening. These findings challenge assumptions that carbohydrate reduction exacerbates disordered eating and suggest that abstinence-based dietary strategies, when embedded within a psychologically supportive framework, may be beneficial for some individuals with BED and UPFA. Controlled trials are warranted to establish efficacy and inform clinical guidelines.

## Introduction

Binge Eating Disorder (BED) is characterised by recurrent episodes of eating objectively large quantities of food in a discrete period, accompanied by a sense of loss of control, distress, and the absence of compensatory behaviours such as purging or excessive exercise (1). BED is the most common eating disorder globally, affecting an estimated 1.9% of the population, with higher prevalence among individuals with BMI ≥ 30 kg/m^2^, depression, and metabolic syndrome (2). Despite the growing recognition of BED in both clinical and public health contexts, treatment options remain limited. Cognitive Behavioural Therapy (CBT) and pharmacotherapy (e.g., lisdexamfetamine) are commonly recommended; however, their long-term efficacy is modest, with high relapse rates and no observable improvements in outcomes over the last four decades (3). One reason may be that first-line treatments for BED generally do not acknowledge the addictive potential of some foods for certain people. Thus, an integration of addiction perspectives may represent a missed opportunity to improve care for a subset of individuals with binge eating patterns.

### The role of ultra-processed foods in BED and UPFA

The NOVA classification system categorises foods on the extent of their processing, from “NOVA-1” minimally processed foods, to “NOVA-4” ultra-processed, containing little to no identifiable NOVA-1 foods. Ultra-processed foods (UPFs), broadly defined under the NOVA classification as industrial formulations high in refined carbohydrates, fats, additives, and flavour enhancers, have been increasingly implicated in patterns of compulsive overeating (4). These foods are engineered for hyperpalatability and have been shown to override satiety mechanisms, promoting excessive consumption and disrupting appetite regulation (5). Neuroimaging studies have indicated that UPFs recruit dopaminergic reward pathways just like other known addictive substances, such as alcohol or nicotine (6, 7).

The construct of Ultra-Processed Food Addiction (UPFA) has emerged as a framework for understanding this compulsive consumption (8). UPFA is characterised by symptoms consistent with substance use disorders, including craving, loss of control, continued use despite harm, and withdrawal-like symptoms, when individuals consume or attempt to abstain from UPFs (9, 10). The Yale Food Addiction Scale (YFAS) has been widely used to operationalise this concept, with prevalence estimates suggesting a significant overlap between UPFA and binge eating behaviours, particularly among individuals with BMIs > 30 kg/m^2^ (11). Although UPFA is not yet formally recognised in diagnostic manuals, its growing empirical support and relevance to BED and other eating disorders warrant further exploration (8). Efforts to disentangle BED from UPFA have identified several shared and unique mechanisms (12). Overlapping causes and consequences include reward dysfunction, craving, emotion dysregulation, and impulsivity. Unique aspects of addictive eating involve the importance of the specific foods (salience), withdrawal symptoms, and escalating tolerance. Unique aspects of BED include dietary restraint, as well as shape and weight concerns (12). The overlap between the constructs has been estimated to be between 42%–57%, suggesting that about half of disordered eaters meet criteria for both, while the other half fit more neatly into one category or the other (13, 14). It has been suggested that many individuals seeking treatment for BED have UPFA symptoms, but this designation is frequently overlooked, leaving many patients frustrated and confused (15, 16). Thus, addiction-informed approaches to the treatment of binge eating remain under-researched and under-utilized.

### Addiction-informed approaches to binge eating and UPFA

Although Liberate was developed to support adults with self-reported UPFA rather than to treat BED specifically, the overlap between UPFA and binge eating made it clinically important to examine changes in binge eating symptoms following participation. Addiction models, such as the incentive sensitisation theory, suggest that repeated exposure to highly rewarding stimuli (e.g., UPFs) sensitises neural pathways that enhance the salience of these stimuli, leading to compulsive use (17, 18). This overlapping pattern with other substance-related disorders has led some researchers and clinicians to propose implementing addiction recovery frameworks, including abstinence, 12-step programmes, and recovery-based peer support in the treatment of UPFA and related binge eating behaviours (19). Early pilot studies have shown promising results in terms of reducing food cravings, improving psychological wellbeing, and promoting weight regulation using these models (20, 21). While UPFA and BED share many neurobiological and behavioural mechanisms, each also has unique aspects that indicate they are not synonymous (12).

### Dietary approaches to binge eating recovery

Traditional eating disorder treatment paradigms caution against the exclusion of food groups, or macronutrients such as carbohydrates, based on the perspective that restriction may increase the risk of bingeing and reinforce disordered eating patterns (22) However, much of this guidance derives from studies involving low-calorie or overly restrictive dieting protocols, which are metabolically and psychologically distinct from low-carbohydrate approaches that emphasise real, satiating foods and are not calorie-deficient. Moreover, low-carbohydrate diets have been shown to stabilise blood glucose, reduce hunger and cravings, and promote satiety, all of which may reduce binge eating urges (23, 24). Furthermore, ketogenic and low-carbohydrate dietary interventions have demonstrated efficacy in improving mood, reducing UPFA symptoms, and supporting remission of binge-eating behaviours in several small studies (25, 26). When implemented within a psychologically supportive, addiction-informed framework, carbohydrate reduction may be complementary to recovery from BED and UPFA, rather than a risk factor for relapse. Some have gone as far as to call the dietary restraint theory of eating disorders a fallacy (27).

### The rationale for the current study

Despite the evidence supporting both addiction psychoeducation and dietary interventions independently, few studies have integrated these approaches in a structured intervention. The Liberate programme was developed to address this gap: an online, group-based, psychoeducational course for individuals with UPFA that incorporates peer support, addiction science, and a real food, low-carbohydrate dietary approach. While the primary aim of the original feasibility study was to assess the acceptability and implementation of Liberate (28), the observed reductions in participants’ binge-eating behaviours prompted further analysis of changes in Binge Eating Scale (BES) scores. Improvements were not only statistically significant post-intervention but also sustained at six-month follow-up, warranting additional reporting.

## Objective

To investigate changes in BES scores following participation in the online UPFA psychoeducation course.

## Methods

### Study design

This paper presents a secondary analysis from a pre-and post-test feasibility study and nested acceptability study of Liberate (published elsewhere (28). Binge eating symptoms were assessed at baseline, post-intervention (8 weeks), and 6-month follow-up.

### Participant eligibility

#### Inclusion criteria

- Aged ≥18 years
- Body mass index (BMI) of >20 kg/m^2^
- Willing and able to provide informed consent to participate and adhere to the study procedures
- Self-reported UPFA
- Able to read and speak English
- Has access to a computer with camera and microphone

#### Exclusion criteria

- In a current episode of anorexia nervosa (e.g., self-imposed anorexia nervosa with rapid weight loss), requiring immediate referral to an eating disorder team.
- Likely to have bariatric surgery in the next 6 months or have had bariatric surgery in the last 12 months, as this would skew the measure of BMI (kg/m^2)^.
- Pregnant or planning to become pregnant in the next 6 months, as this would skew the measure of BMI (kg/m^2)^.

### Recruitment

Participants were recruited through posts from the service provider on social media platforms, including Instagram, X (formerly Twitter), and TikTok. The service provider posted content to targeted social media users who may self-report a UPFA through tagging appropriate people in the field and support organisations. Potential participants were invited to attend live 30-min information sessions in January 2024. Information sessions were held online and repeated at eight different times of the day and week, scheduled in the same time slots as the intervention to confirm accessibility. If subsequently interested in participating, individuals were signposted to the study information sheet, consent forms and screening questionnaire to determine their eligibility. Following the initial feasibility and acceptability study (26), a subsequent cohort, following the same recruitment method and completing the same programme under the same protocol was included to explore whether observed reductions in binge-eating symptoms remained consistent and to strengthen the robustness of findings through an increased sample size.

### Intervention

Liberate is described in more detail in a previous publication. The intervention was reported using the Template for Intervention Description and Replication (TIDieR) checklist and mapped to the Behaviour Change Technique Taxonomy v1 to support transparency and replicability (42, 43). Briefly, Liberate is a community-based intervention for people living with UPFA delivered by a community charity organisation (Public Health Collaboration; Charity No. 1171887). Delivered online, eight coach-led educational sessions delivered over 8 week alongside 12 months of peer-to-peer support. Educational content centres on addiction-informed psychoeducation, including the scientific mechanisms underpinning brain activity in addiction, delivered in an easy-to-understand format that provides recipients with non-judgemental explanations for their addiction-like symptoms. The intervention also incorporates a real-food, low-carbohydrate, abstinence-oriented dietary approach, supported within a psychologically informed and community-based framework. Sessions are delivered by trained coaches, who provide guidance and support throughout the programme.

### Data collection

Participants provided data on their sex, age, ethnicity, and BMI (kg/m^2^.)

#### Outcome

The outcome for this analysis was changes in BES scores. The BES is a 16-item self-report instrument developed by Gormally et al. (29) to assess the presence and severity of binge eating behaviours, particularly among individuals living in larger bodies. The scale assesses both behavioural features (e.g., rapid consumption of substantial amounts of food) and emotional/cognitive dimensions (e.g., feelings of guilt or loss of control). Each item contains multiple response choices scored from 0 to 3, resulting in a total score ranging from 0 to 46. Based on these totals emerges three clinical severity categories: ≤17 indicates little or no binge eating, 18–26 suggests moderate binge eating, and ≥27 is consistent with severe binge eating pathology (29, 30). The BES shows strong psychometric properties, including high internal consistency (Cronbach’s alpha > 0.85) and, although not diagnostic on its own, shows good construct validity with clinical interviews for binge eating disorder (BED) (30). The tool is commonly used in both clinical and research settings to screen for disordered eating and track symptom change. In this study, the BES was completed at baseline, post-intervention, and follow-up. Previous research has demonstrated good internal consistency for the BES scale (Cronbach’s *α* > 0.85) (26). Internal consistency was not recalculated in this analysis as item-level BES data were not available for the full sample (only total scores).

## Data analysis

All quantitative analyses were conducted using SPSS (version 26; IBM Corp., Armonk, NY) (31). This paper reports a secondary analysis of data collected as part of the Liberate feasibility and acceptability study, with additional participants recruited. Data were pooled given consistency in measurement and participant profiles. Descriptive statistics were used to summarise baseline characteristics, and categorical BES changes were reported descriptively (e.g., proportion of participants moving from severe to moderate/mild categories). The primary outcome for this analysis was the change in BES scores across three timepoints: baseline, post-intervention (8 weeks), and six-month follow-up.

BES scores were analysed as both a continuous variable (range: 0–46) and as a categorical measure, using established thresholds for mild/no binge eating (≤17), moderate (12, 18–25), and severe (≥27). Repeated-measures ANOVA was conducted on BES total scores, with a Bonferroni correction. Friedman Test was conducted on BES category data to compare the mean ranks between the timepoints before paired comparisons were completed using Wilcoxon signed-rank tests. An intention-to-treat (ITT) approach was adopted, consistent with the original feasibility study, whereby baseline scores were carried forward for participants with missing post-intervention or follow-up data. Ethics was approved and provided by Coventry university ethics committee: P168471.

## Results

### Participant characteristics

**Figure 1:**
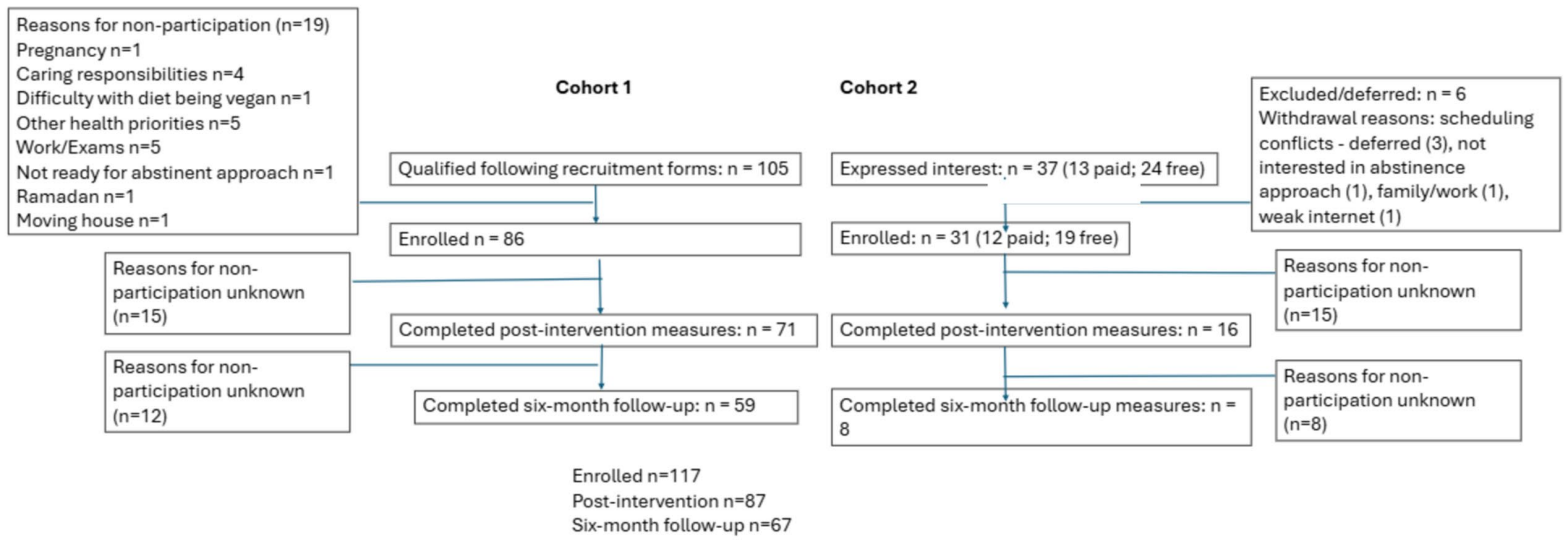
Participant flow across the two Liberate cohorts (CONSORT).

Across the original Liberate programme (28) and a more recent cohort receiving the same programme, there were 117 participants in total. Participants were predominantly female and White, with most aged between 35 and 64 years. BMI categories indicated that the majority of participants were living with overweight or obesity. Full demographic and clinical characteristics are presented in Table 1, Participant flow across the two cohorts is shown in Figure 1.

**Table 1:** Participant characteristics by cohort, including ethnicity, age, sex, and BMI category.

Data received	Cohort 1	Cohort 2
Months recruited at	Feb 2024	May 2024
N	86	31
Ethnicity		
Asian	5	2
Black African Caribbean	1	1
Mixed or other	5	2
White any	75	26
Age range		
18–24	1	0
25–34	16	2
35–44	27	3
45–54	19	11
55–64	16	9
65–74	7	5
75–84	0	1
Sex		
Female	79	24
BMI (kg/m^2^)		
Normal weight	16	2
Pre-obesity	22	7
Obese I	20	3
Obese II	14	6
Obese III	14	13

#### Changes in BES total score

**Figure 2:**
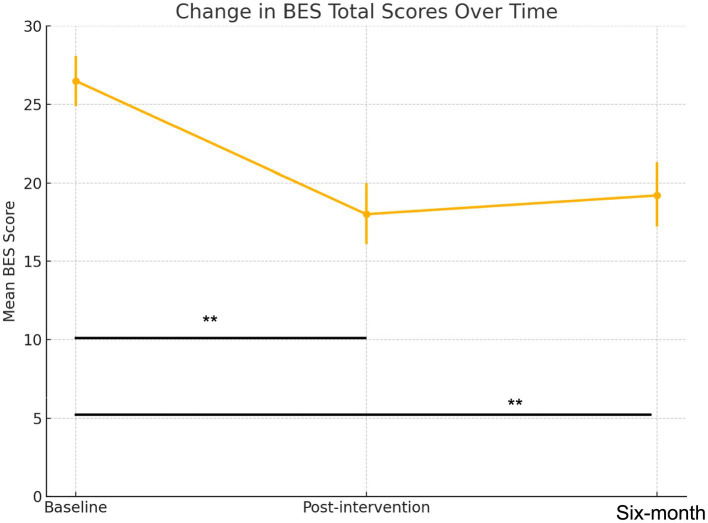
Change in BES scores at baseline, post intervention, and at six-month follow-up; **p = <0.001.

In the BES total score analysis, the average score at baseline was 26.47 (95% CI: 24.87–28.06), consistent with moderate-to-severe binge eating. Scores reduced to an average of 18.04 (95% CI: 16.12–19.96; *p* = <0.001) post-intervention and plateaued at 19.25 (95% CI: 17.21–21.28; *p* = <0.001) at follow-up (Figure 2). The difference between post-intervention and follow-up was not statistically significant (from a score of 18.04 to 19.25, *p* = 0.276).

#### Changes in BES categories

Overall, there was a statistically significant difference in BES categories across the time points (*p* = <0.001).

#### Changes in BES categories: baseline to post-intervention

At post-intervention, the number of participants classified as having no binge eating symptoms more than doubled, from 22 (18.8%) at baseline to 64 (54.7%). Conversely, the number of participants in the “severe” category reduced from 57 participants (48.7%) to 29 (24.8%). Moderate binge eating also declined, from 38 participants (32.5%) to 24 participants (20.5%). Overall, 75 (64%) participants reported a lower severity category post-intervention. These reflected statistically significant differences (*p* ≤ 0.001).

#### Changes in BES categories: baseline to 6-month follow-up

At the six-month follow-up, improvements in binge eating severity were sustained. The number of participants in the “none” category increased from 22 (18.8%) at baseline to 57 (48.7%) at follow-up. The number of participants in the “severe” category increased post-intervention from 29 to 36 (30.8%) at follow-up. This remained below baseline levels, but the difference was statistically significant (*p* ≤ 0.001).

#### Changes in BES categories: post-intervention to 6-month follow-up

**Figure 3:**
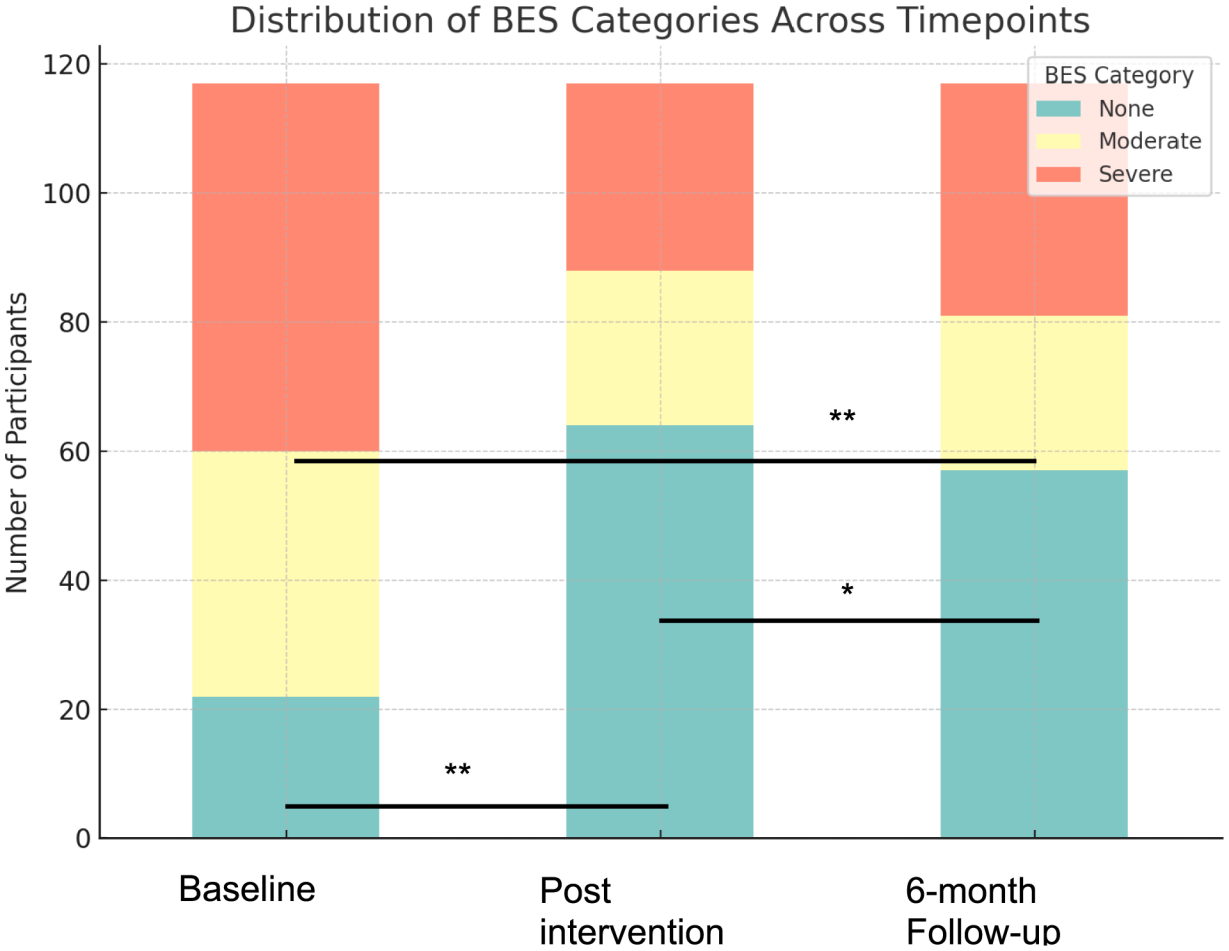
Distribution of BES severity categories at baseline, post-intervention, and six-month follow-up; *p ≤ 0.001 and *p = 0.048.

Between post-intervention and six-month follow-up, changes in binge eating severity classifications were modest. A smaller, but statistically significant, difference was observed (*p* = 0.048). The number of participants in the “none” category decreased from 64 participants (54.7%) to 57 (48.7%). The number of participants in the “severe” category increased from 29 participants (24.8%) to 36 (30.8%). Yet, this remained lower than the baseline figure of 57 (48.7%) participants. Overall, there was small statistically significant difference between these time points (Figure 3).

## Discussion

This secondary analysis set out to examine the impact of the Liberate online intervention, which integrates addiction-informed behavioural principles with a low-carbohydrate dietary approach, on symptoms of binge eating among individuals experiencing UPFA. The findings suggest a reduction in binge eating behaviours following participation in the programme, indicating that addressing UPFA through structured support and dietary modification may offer an effective route for symptom improvement.

Given the established links between UPFA and disordered eating behaviours, particularly binge eating (32, 33), this feasibility and acceptability study explored whether engagement with the Liberate intervention, centred on abstinence from problematic UPF’s and a structured low-carbohydrate diet, was associated with a reduction in binge eating symptoms. While not designed to formally assess efficacy, changes in BES scores were examined to assess whether the intervention had a beneficial, rather than harmful, impact.

UPF’s have been shown to activate reward pathways in the brain in a manner similar to addictive substances, leading to compulsive consumption despite negative consequences (32, 33). Moreover, metabolically, dietary interventions that focus on stabilising blood glucose and eliminating common trigger foods have been associated with improved appetite regulation and reduced food cravings (34, 35). The Liberate programme also incorporates addiction recovery principles, including community support, psychoeducation, and behavioural tools, known to enhance treatment outcomes in both substance use and food-related disorders (19, 20, 36).

### Interpretation of findings

Statistically significant reductions in BES scores were observed from baseline to post-intervention, and these gains were mostly maintained at follow-up, suggesting stable clinical improvement over time. The change in BES total score between post-intervention and six-month follow-up was not statistically significant, indicating that improvements in binge-eating symptom severity were largely sustained after the intervention period. The proportion of participants in the “severe” binge eating category nearly halved post-intervention, and remained 37% lower than baseline at follow-up, highlighting a sustained shift away from the binge eating severity. Similarly, the number of individuals classified as having “none” (or minimal) binge eating symptoms increased substantially and remained mostly elevated at six-month follow-up. We found that binge eating symptoms did not seem to be exacerbated by abstinence from UPF and low carb diet in individuals with UPFA symptoms. These outcomes suggest that the Liberate intervention, and its abstinence, low carb and continuous support approach, may reduce the frequency and severity of binge eating episodes, with the greatest improvements observed shortly after the intervention was completed, with minimal regression over 6 months. This is supported by the findings of substance use disorder treatments, where continuous support is provided for prolonged recovery, prevention of relapse, and harm reduction support (37).

### Comparison with existing literature

Previous studies have found limited long-term success using traditional treatment modalities such as Cognitive Behavioural Therapy (CBT) or pharmacological approaches, which are often associated with relapse or poor accessibility outside specialist care settings (3). In contrast, this study aligns with emerging evidence supporting the use of addiction-informed frameworks to address binge eating (19, 20), as well as small-scale studies indicating benefits from low-carbohydrate or ketogenic diets in reducing binge eating symptoms (38, 39). These approaches may better serve individuals whose eating behaviours reflect addiction-like patterns, such as loss of control, persistent cravings, and withdrawal-like symptoms in response to eliminating highly processed foods (9).

### Mechanisms and theoretical framing

Several mechanisms may underlie the observed improvements. The incentive sensitisation theory suggests that repeated exposure to highly rewarding stimuli, such as ultra-processed foods, enhances the salience of these cues through dopaminergic pathway sensitisation, leading to compulsive overconsumption (18). Abstinence-based strategies, as encouraged in Liberate, may help reduce exposure to these cues, thereby diminishing the reward response over time. Additionally, the dietary component of the intervention likely supported stabilisation of blood glucose and insulin levels, reducing physiological drivers of hunger and cravings (18, 34). The community and psychoeducational elements of Liberate are consistent with recovery models in substance use treatment, promoting self-efficacy, social connection, and behavioural change (19, 20, 40). The combination of these components may be especially effective for individuals with UPFA, whose binge eating behaviours are not solely psychological but also neurobiologically and physiologically mediated.

### Strengths and limitations

The study has several strengths. It was conducted in a community setting, using an online delivery model that enhances accessibility and scalability. The inclusion of a six-month follow-up provides preliminary insight into the longer-term sustainability of symptom changes. The use of validated tools such as the BES allows for comparison with other research and clinical settings. The original mixed-methods study showed the qualitative and quantitative feasibility and acceptability of the Liberate programme, though for this secondary analysis we have reported only the quantitative outcomes for binge eating. An intention-to-treat approach attempted to account for missing data due to attrition, which conservatively assumes there is no improvement in outcomes, however, does not rule out the possibility that symptoms were made worse. An additional strength of this analysis is the supporting evidence of providing behavioural and psychological support alongside dietary changes. The study also has limitations, as a single-arm feasibility study, the lack of a control group means that cause and effect cannot be confirmed. The sample size was not planned in advance to assess changes in binge-eating scores, as the data were collected from a feasibility study. While the number of participants was sufficient to show large improvements in binge-eating symptoms, it may not have been sufficient to detect smaller changes over time or differences between groups. There is a self-report bias also present, as there were no healthcare professionals confirming results objectively, along with self-reported data. There is also a self-selection bias, with participants who were already motivated to change most likely to join. Regarding biases relating to the sample: this sample was predominantly white British, between the age of 35 and 54 years and mostly overweight or above according to BMI (kg/m^2^) which may not reflect the broader population group. Additionally, BES is a self-report measure and may not capture the full complexity of binge eating or UPFA symptoms. The intervention was provided free of charge as part of the feasibility study, which may have influenced uptake, retention, and outcomes. Removal of financial barriers may have increased initial uptake by enabling participation among individuals who would not otherwise have accessed the intervention. However, absence of financial investment may also have reduced perceived value or commitment for some participants, potentially contributing to disengagement or attrition.

### Implications for practice and future research

These findings suggest that addiction-informed interventions incorporating dietary abstinence from UPF’s may offer a promising avenue for individuals with UPFA and binge eating behaviours. The maintenance of BES scores from baseline to follow-up is encouraging, particularly given the high relapse rates typically associated with BED (41). However, further research is needed. Randomised controlled trials are required to establish efficacy and to compare this approach with existing standard treatments among people living with UPFA and explore efficacy in people with binge eating symptoms. In addition, qualitative research could explore participants’ experiences in greater depth, shedding light on mechanisms of change, barriers to engagement, and the sustainability of abstinence.

## Conclusion

This study provides preliminary evidence that the online “Liberate” intervention may support reductions in binge eating symptoms among individuals with UPFA. This study provides preliminary evidence that the online “Liberate” intervention may support reductions in binge eating symptoms among individuals with UPFA. Participants experienced statistically significant improvements in BES scores, with gains sustained at six-month follow-up (*p* = <0.001). Notably, there was no statistically significant change in BES total scores (*p* = 0.276) between post-intervention and follow-up, suggesting that improvements achieved during the intervention were largely maintained over time rather than continuing to increase or returning to baseline levels. These findings challenge assumptions that carbohydrate reduction is harmful in the context of all forms of disordered eating, and instead suggest that such approaches, when including psychological support and an abstinence-focused framework, may be beneficial for a subset of individuals with binge eating symptoms and self-reported UPFA. Importantly we found that, abstinence from UPF’s within this supportive framework was not associated with worsening binge-eating symptoms over 6 months. While the results are encouraging, further research is needed to confirm these outcomes in controlled trials and to better understand the mechanisms through which dietary and behavioural interventions exert their effects. Developing targeted treatment pathways that recognise the role of UPF’s as addictive may be a critical step toward improving care for individuals with BED and co-occurring UPFA.

## Data Availability

The raw data supporting the conclusions of this article will be made available by the authors, without undue reservation.
